# A Current View on Dual-Task Paradigms and Their Limitations to Capture Cognitive Load

**DOI:** 10.3389/fpsyg.2021.648586

**Published:** 2021-05-20

**Authors:** Shirin Esmaeili Bijarsari

**Affiliations:** Department of Educational Psychology, Institute of Psychology, Goethe University Frankfurt, Frankfurt, Germany

**Keywords:** cognitive load, dual task, secondary task, measurement, validity, comparability, cognitive load measurement, taxonomy

## Abstract

Dual-task paradigms encompass a broad range of approaches to measure cognitive load in instructional settings. As a common characteristic, an additional task is implemented alongside a learning task to capture the individual’s unengaged cognitive capacities during the learning process. Measures to determine these capacities are, for instance, reaction times and interval errors on the additional task, while the performance on the learning task is to be maintained. Opposite to retrospectively applied subjective ratings, the continuous assessment within a dual-task paradigm allows to simultaneously monitor changes in the performance related to previously defined tasks. Following the Cognitive Load Theory, these changes in performance correspond to cognitive changes related to the establishment of permanently existing knowledge structures. Yet the current state of research indicates a clear lack of standardization of dual-task paradigms over study settings and task procedures. Typically, dual-task designs are adapted uniquely for each study, albeit with some similarities across different settings and task procedures. These similarities range from the type of modality to the frequency used for the additional task. This results in a lack of validity and comparability between studies due to arbitrarily chosen patterns of frequency without a sound scientific base, potentially confounding variables, or undecided adaptation potentials for future studies. In this paper, the lack of validity and comparability between dual-task settings will be presented, the current taxonomies compared and the future steps for a better standardization and implementation discussed.

## Introduction

Empirical studies in educational research are often accompanied by the term cognitive load and its measurement. As a construct based on the Cognitive Load Theory (Sweller et al., 1998), it is depicted to reflect the utilization of mental resources, in particular the working memory of an individual, *via* their level of exhaustion. It is assumed to vary between a higher or lower state, depending on the tasks performed, for instance, writing an essay versus reciting simple vocabulary. By identifying the parameters exhausting the mental resources, instructional settings can be adapted for a higher learning outcome. For this purpose, different methods to measure cognitive load have been developed over the years. Brünken et al. (2003) classify these methods based on their objectivity and causal relationship into four categories: subjective-direct, subjective-indirect, objective-direct, and objective-indirect methods.

Subjective measurements can be summarized as self-reports like questionnaires (Leppink et al., 2013) to assess the perceived mental effort. It is not a method best used for continuous assessment as it is executed retrospectively (Brünken et al., 2003) and seems to be influenced in the sensitivity and accuracy of its results by the timing and frequency of its use (Chen et al., 2011; van Gog et al., 2012). Nonetheless, it is so far the only method to attempt to identify the cognitive load distinguished by its three dimensions intrinsic, extraneous, and germane load (Brünken et al., 2010; Leppink et al., 2013; Klepsch et al., 2017). In contrast, objective measurements assess the performance of the individual simultaneously to the task and vary from physiological methods like electroencephalography (Antonenko et al., 2010) or fMRI (Whelan, 2007) to dual tasks (Park and Brünken, 2018). Chen et al. (2011) found the objective measurements more lacking compared to subjective measurements, because of their lower sensitivity toward small changes in the cognitive load during a task. Brünken et al. (2003), however, emphasized the difference in accuracy between indirect and direct measurements based on the causal relation of mental effort and experienced cognitive load. In that regard, indirect measurements tend to be unreliable in their interpretation as other factors might have influenced the reported responses (Brünken et al., 2010). Objective-direct measurements like neuroimaging and dual tasks, however, relate directly to the experienced cognitive load (Brünken et al., 2003). And while neuroimaging methods like fMRI seem promising, some limitations arise by the intrusiveness of the technical device. Dual tasks, often also referred to as secondary tasks, present an objective-direct measurement in which two tasks are to be performed simultaneously to observe performance drops in either task. There are two ways to conduct dual tasks, either to induce or to assess cognitive load (Brünken et al., 2002; Klepsch et al., 2017). To induce cognitive load, the secondary task is designed to demand the mental resources needed for the primary task, for instance, by tapping or humming a melody (Park and Brünken 2015; Sun and Shea, 2016). Therefore, the performance of the primary task is affected. In contrast, the cognitive load can also be assessed by simple decision-making tasks like mathematical tasks (Lee et al., 2015; Tang et al., 2015), to observe the performance of the secondary task without influencing the primary task.

Due to these differences in objectivity and causal relation, dual tasks might be seen as an adequate alternative to assess cognitive load as a simultaneous, objective-direct measurement. However, the current state of research showcases a broad variety and heterogeneity of dual-task methods that lack standardization and continuity in their implementation. This in turn hinders the validity and comparability between studies as well as an accurate depiction of the cognitive load throughout the learning process. To further expand on this discrepancy between intent and implementation of dual tasks, this paper will discern the underlying cause of the lack of validity and comparability and present the current state on the taxonomy of dual tasks.

## The Lack of Validity and Comparability in Dual-Task Settings

For a better understanding of the proclaimed issues, the validation as formulated by Kane (2013) should be consulted. He states in his argument-based approach that two steps have to be executed to ensure validity: specifying the proposed interpretation or use of the test and evaluating these claims based on appropriate evidence. The evidence is collected through four inferences that build up from a single observation in a test setting, for instance, a multiple-choice question, to the implementation of the target score as a reflection of the real-life performance. In the dual-task setting, it is comparable to question who and what the task is going to assess, which parameters encompass the proposed interpretation and use and if the determined parameters result in its successful accomplishment. However, aside a few exceptions, there is a lack of empirical investigation of secondary tasks, not only regarding their psychometric properties but also in relation to their respective dual-task settings (Watter et al., 2001; Jaeggi et al., 2010). Contrary to the assumption of validity being universal for every setting of its respective test (Kane, 2013), validity has to be examined for each new proposed interpretation and use. A similar sentiment can be found in the study of Jaeggi et al. (2010), where one of the more common secondary tasks, the n-back task, was examined on its validity. The mixed results showed not only difficulty in confirming its validity but also a further need for implementation and examination in different settings.

Another issue arises in the form of lacking comparability between the different dual-task studies. Currently, most dual tasks are custom-made for their specific instructional setting, without any reference to an evaluated and standardized method. Most often, the decision behind the choice of a dual-task method is not further discussed, which in turn might hinder future researchers in continuing or implementing these studies. The different types of dual task not only lack a framework by which a fitting task can be chosen but they also ignore natural limitations in combining different tasks, for instance, a primary motoric task of walking and a secondary task of typing on a phone. This setting would result in a reduced performance of the primary task as the secondary task is naturally intrusive by limiting the field of vision (Lamberg and Muratori, 2012). Nor do they focus as much on the aspect that experience in multitasking can increase the ability to dual task (Strobach et al., 2015) or that dual tasks are great to measure progress in novices but not experts (Haji et al., 2015). Similarly, to the topic of experts, there can be confounding variables, for instance, response automatization (van Nuland and Rogers, 2016) and age, in particular dementia, influencing the participants (Toosizadeh et al., 2016; Sawami et al., 2017).

## The Current Taxonomy of Dual Tasks

Despite the broad heterogeneity of dual-task methods in instructional settings, one common denominator can be found. A dual-task setting consists of two tasks: the primary task that the researcher wants to observe and the secondary task that has no connection to it beyond its competitive nature. The participant has to perform both tasks concurrently. Apart from that, most attempts at creating a systematic approach toward the variety of dual-task methods have been few and far between and lacking a holistic view.

One of the earlier taxonomies by Brown (1978) postulated four design factors to determine differences between dual-task methods: the information processing demand, the prioritized task performance, the temporal structure and the locus of interference. The first design factor focused on the demand the chosen secondary task puts onto the information processing – either by stimuli with constant or variable demands, for example, changing between easy and complex tasks, or by continuously variable and continuously constant demands not bound to specific stimuli. Another role played the priority given to the secondary task, which could be either primary, secondary, or of equal importance to the primary task. It could be compared to the priorly mentioned ways of inducing or assessing cognitive load (Brünken et al., 2002; Klepsch et al., 2017). van Nuland and Rogers (2016) further recommended the task priority to be explicitly stated in the participants’ instructions, as there otherwise might be a task performance trade-off. The third design factor by Brown (1978) focused on the temporal structure of the secondary task, which was either force-paced by the experimental setting, self-paced by the participant or force-paced by the experimental setting within a specific time interval. Lastly, the locus of interference between both tasks could either be at the sensory input or motor output, within the process of the tasks or a combination of all three. He argued though that both sensory input and motor output should not be used as a locus of interference as the dual-task method intends to focus on the mental resources and therefore needs to be used during the process of the mental activity.

Another attempt at categorizing and standardizing dual tasks from a physician’s viewpoint has been made by McIsaac et al. (2015). Three main categories were stated: tasks by action, task complexity, and task novelty. The category of tasks by action distinguishes between dual tasks consisting of both cognitive, both motor, and cognitive-motor or motor-cognitive primary and secondary task combinations. Therefore, the selection of the proper dual-task method does not only focus on finding a fitting secondary task contentwise but also on its execution in combination with the primary task. The second category, task complexity, is in general a relevant factor but not easy to standardize. The complexity of a task might be felt differently for someone that has never done it versus an experienced user. In this case, task novelty also plays a role as the experience influences the complexity and therefore also the measurement results (Strobach et al., 2015).

Lastly, the recent taxonomy by Wollesen et al. (2019) focused on the different task types. They distinguished between reaction time tasks, controlled processing tasks, visuospatial tasks, mental tracking tasks, working memory tasks, and discrimination tasks. The reaction time tasks were defined as tasks that rely on the reaction time between the sensory stimulus and the behavioral response, for example, pressing a button whenever a light goes on. The controlled processing task expands the reaction time task by the addition of a decision-making process, for example, pressing a button only when a specific symbol appears. The visuospatial task focuses on detecting or processing visual information, for example, finding a symbol in a rotated position. The mental tracking tasks require the memorization of information and are split into two subcategories: the arithmetic tests, for example, counting backward in 3 s (n-back tasks), and the verbal fluency, for example, naming words starting with the same letter. The working memory tasks are a simpler form of the mental tracking tasks as they only require holding information but not processing it, for example, memorizing a picture that has to be found again afterward. Lastly, the discrimination tasks focus on the selective attention toward a specific stimulus, for example, the Go/NoGo tasks in which participants have to either provide or withhold a response depending on the stimulus (Verbruggen and Logan, 2008).

Expanding on the visuospatial tasks presented by Wollesen et al. (2019), a few more modality-related classifications can be found. The method of tapping or humming melodies (Park and Brünken 2015; Sun and Shea, 2016), mathematical tasks (Lee et al., 2015; Tang et al., 2015), and visual tasks like reading text or symbols (Scerbo et al., 2017; Wirzberger et al., 2018) showcase that the modality between primary and secondary task can differ between auditory/vocally, visually, and motoric tasks. Furthermore, as mentioned by Brown (1978) and Wollesen et al. (2019), there can be differences in the frequency of the dual task, from event- or interval-based tasks that appear, for example, every 3, 5, or 7 s to continuous tasks that constantly request the participants’ attention. Yet, there is not really a study to be found that uses dual tasks continuously. Most rely on either interval- or event-based frequency.

## Outlining a Holistic Taxonomy

**Figure 1:**
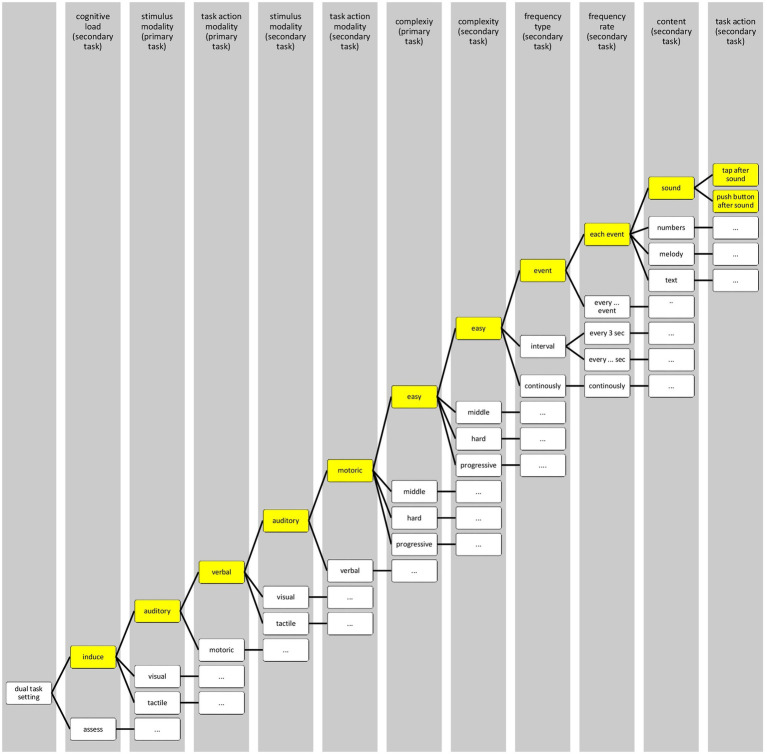
Holistic taxonomy of dual-task settings with exemplary selection paths.

The three taxonomies presented lack a holistic view of the dual-task setting and tend to either simplify or strongly limit the classification. For instance, McIsaac et al. (2015) categorizes tasks by action into cognitive or motor tasks even though the description of detecting a cognitive action outside of an fMRI setting seems contradictory. The participant needs to either act motoric or verbally to respond. In contrast, the taxonomy of Wollesen et al. (2019) expands on the task action by displaying a broader variety of secondary tasks but stays limited to only this one parameter. Furthermore, simply the difference between the two dual-task types of inducing and assessing cognitive load needs to be included in a taxonomy as it changes the intent and therefore the use of it. For this purpose, an attempt at a holistic taxonomy was made (Figure 1).

Parameters relevant to the design of the dual-task setting were included in a stepwise order, ultimately resulting in the selection of the secondary task based on the chosen path. Most of the options are not unique at that, for instance, middle complex tasks can be event-based too. Following the yellow-colored path as an example, after selecting to induce the cognitive load, the stimulus modality and task action modality of the primary task have to be regarded. For instance, choosing a verbal primary task would in turn either hinder a verbal secondary task or restrict the option of higher frequency types in the subsequent parameters. These selections are followed by the complexity of both tasks, and lastly the possible frequency types, frequency rate, and content of the secondary task. Lastly, the task action should show the possible options regarding the prior selections, in this case to either tap or push a button after the sound event, as the secondary task was intended to be auditory in its stimulus but motoric in its action. However, it should be noted that the taxonomy needs to be standardized to be usable as a guide or framework in designing a dual-task setting. The variations of the parameters need to be tested and validated, which, aside from a few exceptions, has yet to be done.

## Discussion

So far, the classifications of the current dual-task paradigms show a mix of different factors without a theoretical framework. Most studies lack a detailed explanation of the reasoning behind the implementation or adaptation of a secondary task, aside the general assumption of using a fitting cognitive load measurement. The presented taxonomies show a broad range of parameters but do not find a common ground. While McIsaac et al. (2015) summarize the different tasks by their action of cognitive versus motoric tasks, the complexity and the novelty of the task, Wollesen et al. (2019) go a bit further and categorize dual tasks by their execution, but with no regards to other parameters. In addition, both taxonomies need to be further specified for a profound framework, especially regarding the different modalities and frequency of dual tasks (Brown, 1978). According to the dual-coding theory (Paivio, 1971, 1991), both verbal information and nonverbal/visual information interact for a better recall, but their information is processed differently in their own channel. Therefore, there should be a higher regard toward the selection of the task modalities and their influence on the cognitive load measurement. Using the same modalities in primary and secondary tasks might contribute to a higher cognitive load measurement because the information is not already distinguished simply by its sensory input. Further influences might be found in the different temporal structure of dual tasks, in particular the frequency in which the secondary task should be used. So far, even empirical studies that describe their task as continuous, end up being high-interval tasks or tasks that cannot be done over a longer time frame because of physical exhaustion, for instance, constant humming or tapping (Park and Brünken 2015; Sun and Shea, 2016). This bears the question on how to change the lack of continuous dual tasks as this particular ability makes it a noteworthy measurement for the cognitive load. Furthermore, it not only needs to be usable over a longer period but also have more variations to be applicable in different settings. For this, it is advisable to look back at the modalities and the restrictions they contain as the physical strain and execution interfere with a continuous dual task. For example, humming a melody might influence an emotional reaction (Schellenberg et al., 2013), but also simply put a physical strain over a longer period. Visual dual tasks would be hard to be kept up in a continuous setting as it would be hard to split the focus of the eyes toward two different tasks, see split-attention effect (Ayres and Cierniak, 2012). A solution might be the use of eye-tracking to adapt the secondary task into a less intrusive method, for example, by changing colors and symbols in the background of the instructional setting to observe the eye movement. In motoric tasks, primary tasks usually cannot be physical as it tends to disturb the secondary task and heightens the physical strain. An exception can be created with physical tasks that work disconnected from each other, for example, tapping on a pedal while sitting and repairing machinery.

Conclusively, future research in relation to dual-task paradigms should take a step back in creating or expanding the different methods of dual tasks and firstly focus on creating a profound and universal taxonomy. Furthermore, the currently existing methods should be evaluated and adapted to create a standardized and reliable use. This of course needs an extensive analysis of the instructional settings and the possibilities to implement dual tasks based on pre-defined variables so that in the future researchers can more easily choose the fitting dual-task paradigms. Dual tasks should furthermore work more toward creating truly continuous tasks to ensure the direct measurement of cognitive load that it proclaims to be (Brünken et al., 2003).

## Data Availability Statement

The original contributions presented in the study are included in the article/supplementary material, and further inquiries can be directed to the corresponding author.

## Author Contributions

The author confirms being the sole contributor of this work and has approved it for publication.

### Conflict of Interest

The author declares that the research was conducted in the absence of any commercial or financial relationships that could be construed as a potential conflict of interest.
